# WCSGNet: a graph neural network approach using weighted cell-specific networks for cell-type annotation in scRNA-seq

**DOI:** 10.3389/fgene.2025.1553352

**Published:** 2025-02-17

**Authors:** Yi-Ran Wang, Pu-Feng Du

**Affiliations:** College of Intelligence and Computing, Tianjin University, Tianjin, China

**Keywords:** scRNA-seq, cell-type annotation, gene expression, graph neural networks, cell specific gene association network

## Abstract

Single-cell RNA sequencing (scRNA-seq) has emerged as a powerful tool for understanding cellular heterogeneity, providing unprecedented resolution in molecular regulation analysis. Existing supervised learning approaches for cell type annotation primarily utilize gene expression profiles from scRNA-seq data. Although some methods incorporated gene interaction network information, they fail to use cell-specific gene association networks. This limitation overlooks the unique gene interaction patterns within individual cells, potentially compromising the accuracy of cell type classification. We introduce WCSGNet, a graph neural network-based algorithm for automatic cell-type annotation that leverages Weighted Cell-Specific Networks (WCSNs). These networks are constructed based on highly variable genes and inherently capture both gene expression patterns and gene association network structure features. Extensive experimental validation demonstrates that WCSGNet consistently achieves superior cell type classification performance, ranking among the top-performing methods while maintaining robust stability across diverse datasets. Notably, WCSGNet exhibits a distinct advantage in handling imbalanced datasets, outperforming existing methods in these challenging scenarios. All datasets and codes for reproducing this work were deposited in a GitHub repository (https://github.com/Yi-ellen/WCSGNet).

## 1 Introduction

Single-cell RNA sequencing (scRNA-seq) is a high-throughput and highly sensitive technology that allows for transcriptome analysis at the individual cell level, significantly enhancing our understanding of cellular heterogeneity and molecular regulatory mechanisms (Eberwine et al., 2014; Kolodziejczyk et al., 2015; Stegle et al., 2015; Potter, 2018). scRNA-seq analysis consists of two main stages: pre-processing and downstream analysis (Luecken and Theis, 2019). The pre-processing stage addresses data quality and variability through steps such as quality control, normalization, batch-effect correction (Tran et al., 2020), feature selection (Deng et al., 2023), and dimensionality reduction (Koch et al., 2021). Downstream analysis then focuses on extracting biological insights, including cell clustering (Petegrosso et al., 2020), pseudotime trajectory inference (Saelens et al., 2019), cell type annotation (Cheng et al., 2023), and differential expression analysis. As scRNA-seq datasets accumulate rapidly, accurate and efficient automatic cell-type annotation have become a crucial step for downstream analyses. It becomes an important approach to a deeper understanding of cellular composition and phenotypic heterogeneity in complex biological systems and diseases (Shao et al., 2021; Jia et al., 2023; Xu et al., 2024).

Traditional cell type annotation primarily relies on manual methods. Experts use known marker genes and related literatures to accurately identify cell types (Clarke et al., 2021; Chen et al., 2023). However, as the volume of data grows rapidly, manual annotation has become increasingly time-consuming and laborious. Moreover, it is highly dependent on expert knowledge and may provide a subjective result (Huang and Zhang, 2021).

As a result, automatic cell-type annotation methods have been developed rapidly. They generally fall into three categories: marker gene database-based, correlation-based, and supervised classification-based (Pasquini et al., 2021; Cheng et al., 2023). Marker gene database-based methods, like scType (Ianevski et al., 2022) and scCATCH (Shao et al., 2020), typically start by clustering cells into distinct groups, followed by using marker gene databases, such as CellMarker (Zhang et al., 2019) and PanglaoDB (Franzén et al., 2019), to identify relevant marker genes. Feature gene selection (Deng et al., 2023) can be applied to refine cell clustering by identifying genes that are most critical for distinguishing clusters, thereby enhancing both resolution and biological relevance. The expression levels of these marker genes within each cluster are subsequently analyzed to map the clusters to their corresponding cell types (Jia et al., 2023). Correlation-based cell-type annotation methods rely on statistical correlations to analyze gene expression data. They automatically compare unlabeled datasets with reference datasets (Pasquini et al., 2021). In contrast to methods that rely solely on marker gene scoring, correlation-based approaches calculate the expression levels of gene sets or entire transcriptomes, thereby enabling a more precise assessment of similarities between datasets (Ranjan et al., 2021). For example, SingleR (Aran et al., 2019) calculates the correlation between a cell’s gene expression and reference cell types to iteratively selecting the optimal gene set to accurately distinguish the most similar cell types. CHETAH (de Kanter et al., 2019) is another correlation-based tool that employs a hierarchical classification approach to annotate cell types.

Supervised classification-based methods train classification models on reference datasets to label cell types in unlabeled datasets. Traditional machine learning algorithms, such as SVM, LDA, NMC, and Random Forest (RF) have been applied in this field (Pedregosa et al., 2011; Abdelaal et al., 2019). Recently, deep learning approaches have also been increasingly adopted. For example, ACTINN (Ma and Pellegrini, 2020) employs a neural network model to learn patterns from gene expression data for cell type annotation. CIForm (Xu et al., 2023) leverages expression data from highly variable genes, using a Transformer architecture to predict cell types based on these features. scDeepInsight (Jia et al., 2023) generates *t*-SNE feature images based on reference datasets to train a CNN for cell type prediction. However, these methods primarily rely on gene expression information and do not fully leverage gene association information. Consequently, graph representation learning has increasingly been applied in cell type annotation research. For instance, scGraph (Yin et al., 2022) utilizes graph neural networks to integrate gene association information, thereby enhancing cell type recognition performance. scPriorGraph (Cao et al., 2024) introduces a dual-channel graph neural network that combines multi-level gene bio-semantics to effectively aggregate feature values of similar cells, achieving efficient cell classification. Beyond cell type annotation, scGNN (Wang et al., 2021) leverages graph neural networks to integrate cell–cell relationships and gene regulatory signals, achieving strong performance in gene imputation, cell clustering, and complex disease analysis like Alzheimer’s. DeepMAPS (Ma et al., 2023) uses a heterogeneous graph transformer to infer cell-type-specific biological networks from scMulti-omics data, integrating cells and genes into a unified graph.

Existing supervised learning methods have yet to incorporate cell-specific networks (CSN) in cell type annotation. CSN is an innovative approach based on scRNA-seq data that constructs a unique gene association network for each cell (Dai et al., 2020; Dai et al., 2019). Traditional methods for gene association network construction typically infer a single network from grouped cell expression data. Among these, WGCNA employs weighted correlation network analysis to construct weighted gene co-expression networks (Langfelder and Horvath, 2008). PCA-PMI utilizes the PC algorithm (Zhang et al., 2012), combined with Part Mutual Information to construct network structures by accurately quantifying nonlinear direct dependencies among genes (Zhao et al., 2016). GRNBoost2 employs gradient boosting within the GENIE3 (Huynh-Thu et al., 2010) framework to infer gene regulatory networks by predicting target gene expression based on the importance of input genes in regression models (Moerman et al., 2019). In contrast to these methods, CSN captures the characterization of individual cellular states and preserves heterogeneity. The network of a cell provides a more reliable representation of its biological system or state (Dai et al., 2019; Li et al., 2021; Wang et al., 2023). Gene interaction strength is related to cellular functions and varies across different cell types. Highly variable genes, which exhibit significant expression differences across cell types, provide valuable information for classification. By integrating the expression profiles and interaction networks of these genes, we can more accurately characterize cell-specific features. In this context, we propose WCSGNet, a graph neural network-based computational approach that utilizes cell-specific interaction networks for automatic cell type annotation. Firstly, highly variable genes are selected. Next, a weighted cell-specific network (WCSN) is constructed based on their expression data to capture gene interaction strengths. This is achieved through an improved CSN construction method (Dai et al., 2019). Finally, a graph neural network is employed to extract features from the WCSN, enabling accurate cell type annotation.

## 2 Materials and methods

### 2.1 Dataset curations

We curated nine benchmarking scRNA-seq datasets, encompassing two species (human and mouse) and three tissue types: pancreas, brain, and peripheral blood. We also applied a comprehensive single-cell atlas for the mouse. The datasets were generated using four sequencing platforms: inDrop, Smart-Seq2, CEL-seq2, and 10X Genomics. The pancreas datasets come from four studies: Baron H. et al. (2016), Baron M. et al. (2016), Muraro et al. (2016), and Segerstolpe et al. (2016). The peripheral blood datasets comprise Zheng 68k (Zheng et al., 2017) and Kang et al. (2018). The Zhang T dataset comes from peripheral blood, normal colorectal, and tumor tissue samples (Zhang et al., 2018). The mouse brain dataset comes from the AMB dataset (Tasic et al., 2018), while the comprehensive mouse cell atlas is the Tabula Muris (TM) dataset (Tabula Muris Consortium, 2018). Among these, the Muraro, Segerstolpe, Zheng 68k, Baron, AMB, and TM datasets are available for direct download from Zenodo (https://doi.org/10.5281/zenodo.3357167). The Zhang T dataset (GEO accession: GSE108989) and the Kang dataset (GEO accession: GSE96583) were obtained from the Gene Expression Omnibus (GEO) database. A detailed summary of the datasets is provided in Table 1.

**TABLE 1:** An overview of the data set used in this study.

Dataset	Tissue	# cell type	# cell	# gene	Protocol	Accession ID
Baron (Human)	Human pancreas	14	8569	17499	inDrop	GSE84133
Baron (Mouse)	Mouse pancreas	13	1886	14861	inDrop	GSE84133
Muraro	Human pancreas	9	2122	18915	CEL-Seq2	GSE85241
Segerstolpe	Human pancreas	12	2133	22757	Smart-Seq2	E-MTAB-5061
AMB	Mouse Brain	4	12832	42625	Smart-Seq2	GSE115746
TM^a^	Mouse	55	54865	19791	10X Genomics	GSE109774
Zheng 68k	Human PBMC	11	65943	20387	10X Genomics	SRP073767
Zhang T	Human PBMC	20	8530	23459	Smart-Seq2	GSE108989
Kang	Human PBMC	8	14617	35635	10X	GSE96583

^a^ TM, tabula muris.

For each dataset, we first filter out cell types with fewer than 10 cells and cells with ambiguous annotations. Next, we remove genes expressed in fewer than 10 cells. Subsequently, we normalize each cell’s gene expression data by dividing each gene’s expression level by the cell’s total expression and scaling by a factor of 10^6^ (Luecken and Theis, 2019).

Let **E** be the gene expression matrix after normalization, we have $E=\left\{e_{i,j}\right\}{\;}_{n\times m}\in R^{n\times m}$ , where *n* is the number of cells and *m* the initial number of genes. We applied the log transformation on each element of the matrix **E** to generate a transformed matrix $E^{\prime }=\{e_{i,j}^{\prime }\}{\;}_{n\times m}\in R^{n\times m}$ , as shown in Equation 1.

$$e_{i,j}^{\prime }\;=\ln\left(e_{i,j}+\varepsilon +1\right),$$ (1)

where *ε* ≥ 0 is a regularization factor. We used the scanpy package (Wolf et al., 2018) to select top *p* highly variable genes (HVGs) from **E**′. The remaining part of **E**′ is denoted as $E_{0}={\left\{e_{0,i,j}\right\}}_{n\times p}\in R^{n\times p}$ , which corresponds to the data matrix consisting of the selected HVGs.

### 2.2 Overview of WCSGNet

**FIGURE 1:**
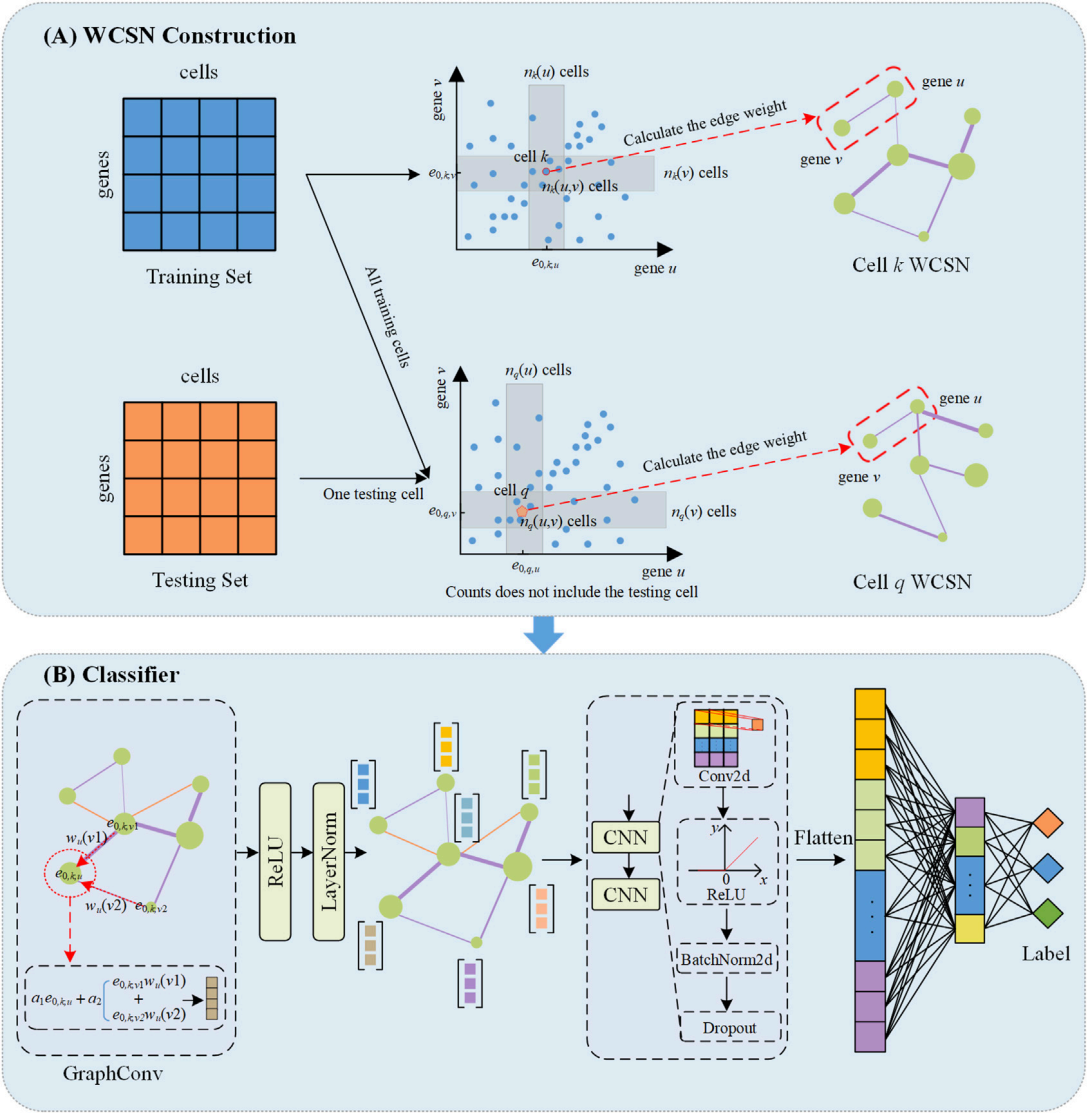
The schematic overview of WCSGNet. **(A)** Construction of weighted cell-specific gene association networks (WCSN). For the training set, WCSN is constructed based on independence tests among genes within the dataset. For the testing set, the construction of WCSN for each cell is based on the gene expression of the current cell and the training dataset. *e*_0,*k*,*u*_ the expression value of the *u*-th gene in the *k*-th cell. Each gray area represents the neighborhood range of gene expression corresponding to the current cell. **(B)** The structure of the classifier. The GraphConv layer aggregates features of the current gene node with the interaction features of its neighboring nodes to generate an updated gene embedding. This is followed by Layer Normalization (LayerNorm) and a ReLU activation function. The processed embeddings are then passed through two convolutional layers (CNN) which include Conv2d, ReLU activation function, BatchNorm2d and Dropout. The CNN output is flattened, and two fully connected layers are subsequently applied to extract higher-level features, ultimately predicting the cell type labels.

WCSGNet is a deep learning model consisting of two modules, as depicted in Figure 1, including the weighted cell-specific gene association networks, and a classifier based on a graph convolutional network. The model takes only scRNA-seq datasets as the inputs to annotate cell types.

### 2.3 Construction of WCSN

We constructed WCSN based on **E**_0_, using an algorithm which is derived from a literature (Dai et al., 2019). Given the *u*-th gene and *v*-th gene in the *k*-th cell, we have the expression value of these two genes as *e*_0,*k*,*u*_ and *e*_0,*k*,*v*_. As in Figure 1A, we have two ranges R_*u*_ ⊆ R and R_*v*_ ⊆ R, which satisfy (*e*_0,*k*,*u*_, *e*_0,*k*,*v*_)∈R_*u*_ × R_*v*_. We then calculate the number of neighboring cells of the *k*-th cell regarding the *u*-th gene and the *v*-th gene, as shown in Equations 2, 3.

$$n_{k}\left(u\right)=\#\left\{i|e_{0,i,u}\in R_{u}\right\}$$ (2)

$$n_{k}\left(v\right)=\#\left\{i|e_{0,i,v}\in R_{v}\right\}$$ (3)

where # is the cardinal operator in the set theory. We calculate the marginal frequencies of cells that have similar expression values, as shown in Equations 4, 5.

$$f_{k}\left(u\right)=n_{k}\left(u\right)/n$$ (4)

$$f_{k}\left(v\right)=n_{k}\left(v\right)/n$$ (5)

Similarly, we can calculate the joint frequency of cells when both the *u*-th gene and the *v*-th gene are considered, as shown in Equations 6, 7.

$$f_{k}\left(u,v\right)=n_{k}\left(u,v\right)/n$$ (6)

where

$$n_{k}\left(u,v\right)=\#\left\{i|\left(e_{0,i,u},e_{0,i,v}\right)\in R_{u}\times R_{v}\right\}$$ (7)

The difference between *f*_*k*_ (*u*,*v*) and the product of *f*_*k*_(*u*) and *f*_*k*_(*v*) represents the statistical relationship between the *u*-th gene and the *v*-th gene in the *k*-the cell, as shown in Equation 8.

$$\rho_{k}\left(u,v\right)=f_{k}\left(u,v\right)-f_{k}\left(u\right)f_{k}\left(v\right)$$ (8)

According to literature (Dai et al., 2019), the *ρ*_*k*_ (*u*, *v*) approximately follows a normal distribution *N* (0, *σ*_*k*_ (*u*, *v*)) when *u* and *v* are independently expressed, where *σ*_*k*_ (*u*, *v*) is shown as Equation 9.

$$\sigma_{k}\left(u,v\right)=\sqrt{n_{k}\left(u\right)n_{k}\left(v\right)\left[n-n_{k}\left(u\right)\right]\left[n-n_{k}\left(v\right)\right]n^{-4}{\left(n-1\right)}^{-1}}$$ (9)

We calculate the normalized *ρ*_*k*_ (*u*, *v*) as shown in Equation 10:

$$\rho_{k}^{\prime }\left(u,v\right)=\frac{\rho_{k}\left(u,v\right)}{\sigma_{k}\left(u,v\right)}=\frac{\sqrt{n-1}\left(n\cdot n_{k}\left(u,v\right)-n_{k}\left(u\right)n_{k}\left(v\right)\right)}{\sqrt{n_{k}\left(u\right)n_{k}\left(v\right)\left(n-n_{k}\left(u\right)\right)\left(n-n_{k}\left(v\right)\right)}}$$ (10)

For computation, we adjusted R_*u*_ and R_*v*_ to cover a fixed proportion of cells that are nearest neighbors of the *k*-th cell. Essentially, this equals to fix *n*_*k*_(*u*) = *n*_*k*_(*v*) = 0.1*n*. We applied one-sided z-test to test every gene pair (*u*, *v*) for *ρ*′_*k*_ (*u*, *v*) in the *k*-th cell. If *ρ*′_*k*_ (*u*, *v*) is large enough to produce a *p*-value less than the threshold *α* = 0.01, the *u*-th gene and the *v*-th gene are associated with a weight *ρ′*_*k*_ (*u*, *v*) in the *k*-th cell.

### 2.4 Classifier based on graph convolution neural network

We applied the GraphConv package (Morris et al., 2019) to aggregate the gene expression value and their associations, as shown in Equation 11:

$$h_{d+1}\left(v\right)=A_{1}h_{d}\left(v\right)+A_{2}\sum_{j\in N\left(v\right)}w_{v}\left(j\right)h_{d}\left(j\right)$$ (11)

where **h**_*d*_(*v*) is the *d*-th layer representation of the *v*-th gene, *N*(*v*) the neighboring nodes in WCSN of the *v*-th gene, *w*_*v*_(*j*) a serial of weights in aggregating gene representations, and **A**_1_ and **A**_2_ trainable parameters. After graph convolution, each gene is represented as a 16-D feature vector. We applied two 2-D convolutional layers, the output channels of these two layers are 12 and 4, respectively. After that, all gene representations are flattened. A MLP was trained on the flattened features to produce cell-type annotations. Figure 1 presents the detail design of the classifier.

### 2.5 Performance estimation

The benchmarking dataset was balanced by up-sampling. If the size of a cell type is less than 5% of the largest cell type. The cells of this type are randomly duplicated so that the size of the type is at least 5% of the largest cell type. We used 5-fold cross-validation to estimate the predictive performance of our method. To minimize the risk of information leak, the partition of training and testing happens before the construction of WCSN. Each testing cell was supplied to the training set individually to construct its WCSN only, while the WCSNs of all training cells are constructed without any information from the testing set. We applied Kaiming Normal Initialization (He et al., 2015) for parameter initialization, with the weighted cross-entropy loss function as shown in Equations 12–14:

$$\left.L=-\frac{1}{n}\sum_{i=1}^{n}\sum_{j=1}^{c}b_{j}[y_{i,j}\log\left(p_{i,j}\right)+\left(1-y_{i,j}\right)\log\left(1-p_{i,j}\right)\right]$$ (12)

where

$$b_{j}=\frac{\min\left(\max\left(b_{j}^{\prime },1\right),50\right)}{\sum_{t=1}^{c}\min\left(\max\left(b_{t}^{\prime },1\right),50\right)}$$ (13)

$$b_{j}^{\prime }=\frac{\max\left(n\left(t\right)|t\in \left(1,...,c\right)\right)}{n\left(j\right)}$$ (14)

*n*(*j*) the number of the *j*-th type cells, *n* the number of all cells, *c* the number of all possible cell types, *b*_*j*_ the weight of the *j*-th type, *y*_*i*,*j*_ a binary indicator that the *i*-th cell belongs to the *j*-th type, *p*_*i*,*j*_ the probability that the *i*-th cell is predicted as the *j*-th type.

### 2.6 Performance measures

To evaluate the predictive performance, accuracy and mean F1-Score were utilized as performance measures. Accuracy is defined as the ratio of correct predictions made by the model to the total number of predictions, as shown in Equation 15.

$$Accuracy=\frac{TP+TN}{TP+TN+FP+FN}$$ (15)

where *TP*, *TN*, *FP* and *FN* are the numbers of true positives, true negatives, false positives, and false negatives. Mean F1-score is calculated by averaging the F1-scores across all cell types, as shown in Equations 16–19.

$$\text{mean}-F1=\frac{1}{c}\sum_{k=1}^{c}F1_{k}$$ (16)

where

$$F1_{k}=2\cdot \frac{precision_{k}\cdot recall_{k}}{precision_{k}+recall_{k}}$$ (17)

$$precision_{k}=\frac{TP_{k}}{TP_{k}+FP_{k}}$$ (18)

$$recall_{k}=\frac{TP_{k}}{TP_{k}+FN_{k}}$$ (19)

*TP*_*k*_, *TN*_*k*_, *FP*_*k*_ and *FN*_*k*_ the numbers of true positives, true negatives, false positives, and false negatives for the *k*-th cell type, and *c* the number of cell types.

### 2.7 Parameter settings

The parameters in our study are set as follows: *ε* = 0 when constructing WCSN and *ε* = 10^–5^ when extracting gene expression features and selection HVGs. This ensures that the gene expression features are not zero, mitigating dropout effects caused by sequencing errors and retaining certain gene expression features. For the construction of the WCSN, we adopted the data processing approach outlined in the CSN paper (Dai et al., 2019) to ensure that genes with an original expression value of 0 remain 0 after log transformation. If a gene pair contains a zero expression value, the edge between them is considered nonexistent (Dai et al., 2019). This computational approach, as set in CSN, aligns with the previously mentioned setting of *ε* = 0, ensuring consistency. Specifically, if a gene pair includes a gene with an expression value of 0, it indicates that there is no association between these two genes (Dai et al., 2019). As for extracting gene expression features, the log transformation is based on the method outlined in the scGraph paper (Yin et al., 2022), which enhances computational stability. We set *p* = 2000 when selecting HVGs. The kernel size and stride of the two 2-D convolutional layers are set to (1, 1) and 1, respectively. The MLP in the classifier contains two hidden layers with 256 and 64 neurons, respectively. We applied Adam optimizer with an initial learning rate of 0.01 and incorporates a weight decay. An Exponential Learning Rate Scheduler (ExponentialLR) (Li and Arora, 2019) with a decay factor *γ* = 0.8 is applied to gradually decrease the learning rate during training, aiding the model for stable convergence. The number of training epochs is set to 30, and the weight decay 10^–4^.

## 3 Results

### 3.1 Performance analysis and comparison

We compared the performance of WCSGNet with 8 state-of-the-art methods across 9 datasets using 5-fold cross-validations. A fixed data split was used for all datasets, and the data splits are available on GitHub repository (https://github.com/Yi-ellen/WCSGNet). The experiments were conducted with a single round of cross-validation using this fixed split, ensuring the reproducibility of the results. The performance values, in terms of mean F1 and accuracy, are listed in Tables 2, 3. The 8 methods in comparison are LDA (Pedregosa et al., 2011), NMC(Pedregosa et al., 2011), RF (Pedregosa et al., 2011), SVM(Pedregosa et al., 2011), SingleR (Aran et al., 2019), CHETAH (de Kanter et al., 2019), ACTINN(Ma and Pellegrini, 2020), and scGraph (Yin et al., 2022).

**TABLE 2:** Benchmark results on nine different scRNA-seq datasets in terms of mean F1.

Method	Zhang T^a^	Kang^a^	Zheng 68k^a^	Baron human^a^	Muraro	Segerstolpe	AMB^a^	TM^a^	Baron mouse
LDA	0.757	0.633	0.556	0.940	0.964	0.987	0.858	0.873	0.895
NMC	0.722	0.753	0.527	0.836	0.763	0.930	0.949	0.745	0.922
RF	0.562	0.727	0.495	0.788	0.963	0.989	0.906	0.803	0.773
SVM	0.805	0.853	0.558	0.967	**0.970**	**0.998**	0.967	0.910	**0.980**
SingleR	0.746	0.767	0.517	0.953	0.953	0.997	0.920	0.809	0.914
CHETAH	0.695	0.677	0.338	0.927	0.938	0.968	0.934	0.789	0.880
ACTINN	0.741	0.843	0.623	0.904	0.970	0.996	0.965	0.886	0.894
scGraph	**0.839**	**0.877**	0.681	0.969	0.961	0.984	0.976	0.921	0.950
WCSGNet	0.768	0.865	**0.703**	**0.978**	0.966	0.993	**1.000**	**0.927**	0.972

^a^ The mean F1 of the baseline methods across these six datasets are derived from scGraph (Yin et al., 2022).

Note: The best results for each dataset are shown in bold.

**TABLE 3:** Benchmark results on nine different scRNA-seq datasets in terms of accuracy.

Methods	Zhang T^a^	Kang^a^	Zheng 68k^a^	Baron human^a^	Muraro	Segerstolpe	AMB^a^	TM^a^	Baron mouse
LDA	0.813	0.743	0.662	0.978	0.970	0.991	0.901	0.954	0.940
NMC	0.769	0.881	0.597	0.912	0.758	0.958	0.976	0.854	0.960
RF	0.718	0.884	0.674	0.962	0.973	0.992	0.985	0.949	0.953
SVM	**0.862**	0.929	0.701	0.986	**0.977**	**0.998**	0.992	**0.977**	**0.984**
SingleR	0.790	0.879	0.673	0.968	0.962	0.997	0.962	0.889	0.910
CHETAH	0.717	0.674	0.298	0.925	0.927	0.955	0.939	0.850	0.895
ACTINN	0.662	0.881	0.468	0.953	0.976	0.996	0.857	0.761	0.967
scGraph	0.834	0.926	0.729	0.983	0.971	0.992	0.991	0.973	0.974
WCSGNet	0.822	**0.939**	**0.765**	**0.987**	0.973	0.994	**1.000**	0.957	0.981

^a^ The accuracy of the baseline methods across these six datasets are derived from scGraph (Yin et al., 2022).

Note: The best results for each dataset are shown in bold.

**FIGURE 2:**
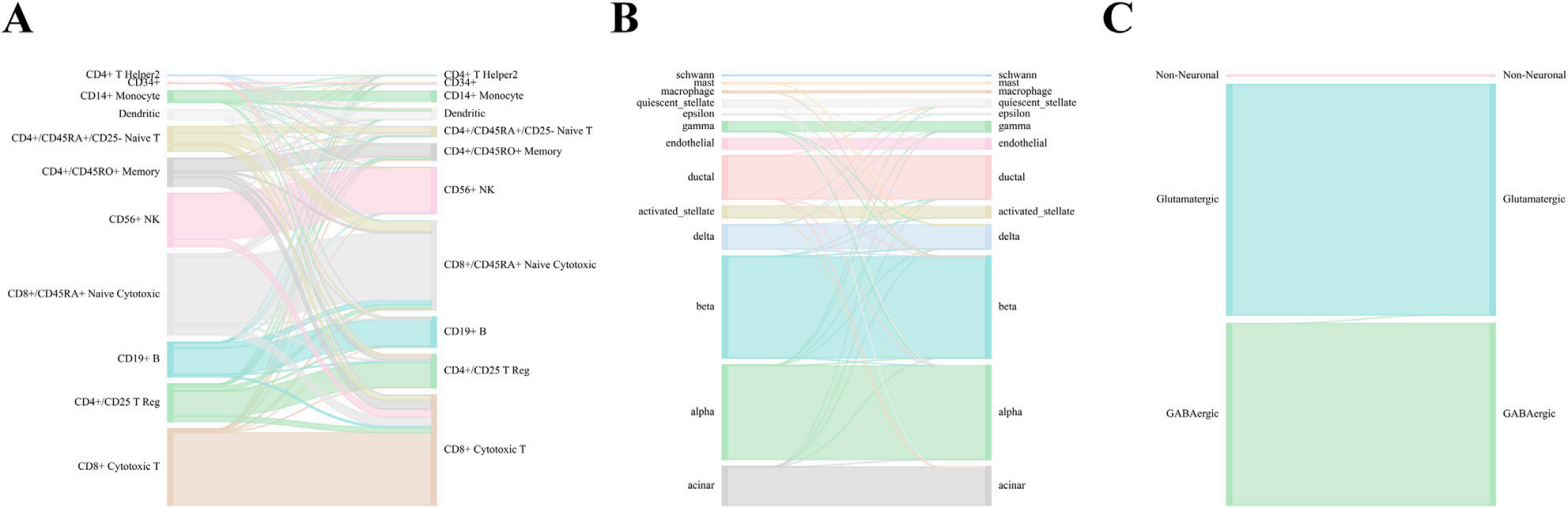
WCSGNet performance across Zheng 68k, Baron Human, and AMB Datasets. Sankey diagrams illustrating the performance of WCSGNet’s 5-fold cross-validation on **(A)** Zheng 68k, **(B)** Baron Human dataset, and **(C)** AMB dataset. The left side represents predicted cell types, while the right side denotes the true cell types. The width of the flows corresponds to the number of cells, providing a visual representation of accurate classification across both major and minor cell types.

In the comparisons, WCSGNet achieves comparable or better performance than other methods. WCSGNet consistently ranks among the leading methods across all benchmarking datasets. WCSGNet achieved the best mean F1 on 4 of 9 datasets (Zheng 68k, Baron Human, AMB, TM) and the second to the best mean F1 on two datasets (Kang, Baron Mouse). It also achieved the best accuracy on 4 of 9 datasets (Kang, Zheng 68k, Baron Human, AMB), and second to the best accuracy on the Baron Mouse dataset. In particular, WCSGNet demonstrated consistently superior performance on the Zheng 68k, Baron Human and AMB datasets. Although the cell type distributions are highly imbalanced, the details of the results (Figures 2A–C) support that WCSGNet has an expectable stable performance.

WCSGNet demonstrated strong classification performance even on imbalanced datasets. The degree of dataset imbalance was assessed using the Imbalance Degree metric (Jia et al., 2024), as shown in Supplementary Table S1. Among the datasets, AMB, Baron Mouse, and Baron Human exhibited the highest levels of imbalance. On the AMB dataset, WCSGNet achieved a mean F1-score improvement of 2.46%, 3.41%, and 3.63% over the top three existing methods (scGraph, SVM, and ACTINN), respectively. Similarly, on the Baron Human dataset, WCSGNet surpassed the top three methods (scGraph, SVM, and SingleR) by 0.93%, 1.14%, and 2.62% in mean F1-score. For the Baron Mouse dataset, WCSGNet’s mean F1-score was comparable to the highest-performing method (SVM), with only a marginal difference of 0.008.

**FIGURE 3:**
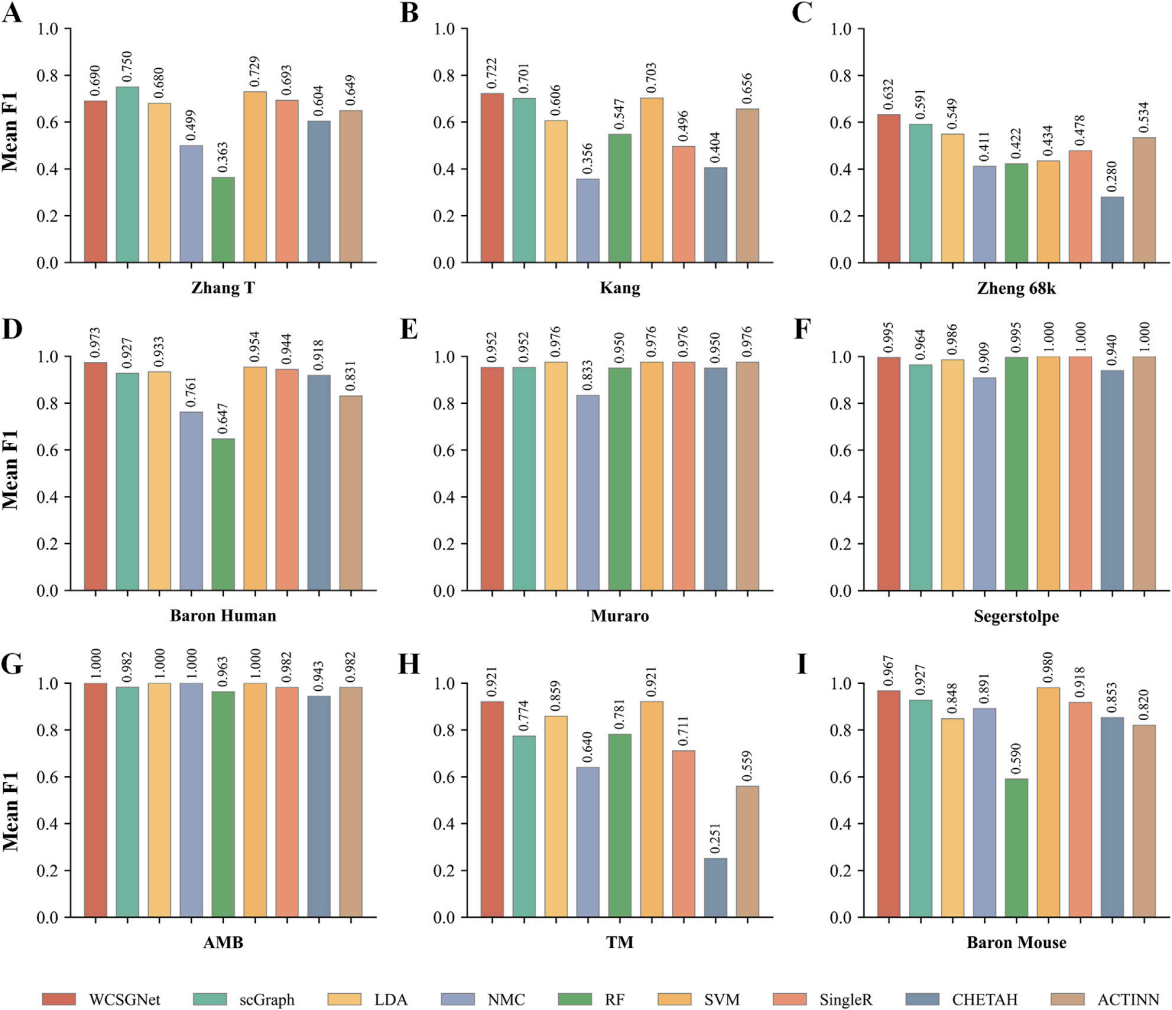
Comparison of rare cell type identification performance across nine scRNA-seq datasets. **(A)** Zhang T dataset, **(B)** Kang dataset, **(C)** Zheng68k dataset, **(D)** Baron Human dataset, **(E)** Muraro dataset, **(F)** Segerstolpe dataset, **(G)** AMB datcaset, **(H)** TM dataset, **(I)** Baron Mouse dataset. Each panel presents a bar chart showing the mean F1-score for WCSGNet and eight baseline methods.

To further analyze the classification performance for each cell type, we conducted experiments across all datasets using various methods, obtaining the F1-score for each cell type, as detailed in Supplementary Table S2. In addition, we examined the recognition performance for rare cell types, defined as those constituting less than 3% of the total cells in the dataset (Wang et al., 2024). As shown in Figure 3, WCSGNet achieved the highest mean F1-score on five out of nine datasets (Kang, Baron Human, AMB) and the second-best mean F1-score on the Baron Mouse dataset. Notably, WCSGNet delivered superior performance in identifying rare cell types across almost all datasets, achieving average improvements in mean F1-score of 1.99% and 3.69% compared to the top two existing methods (SVM and scGraph), respectively.

In addition to its performance on imbalanced datasets, WCSGNet excels in handling large and complex cell datasets. On the TM dataset, which contains 55 cell types and 54,865 cells (Supplementary Table S3), WCSGNet’s mean F1-score surpasses the top three existing methods (scGraph, SVM, ACTINN) by 0.65%, 1.87%, and 4.63%, respectively. Similarly, on the Zheng 68k dataset, which contains 65,943 cells, WCSGNet achieves remarkable improvements in mean F1-scores. It outperforms the top three existing methods (scGraph, ACTINN, SVM) by 3.23%, 12.84%, and 25.99%. On smaller datasets with fewer cell types, like the Muraro datset, WCSGNet still has a good performance, ranking top-three among the 9 methods in comparison.

### 3.2 Analysis of different gene association network construction methods

We compared WCSGNet with different gene association network construction methods, including WGCNA, PCA-PMI, and GRNBoost2, using five-fold cross-validation. Both WGCNA and PCA-PMI generate a symmetric weighted network for each training set, while GRNBoost2 produces an asymmetric weighted network for each training set. The unified network generated from the training set is used for prediction on the test set cells. WGCNA is implemented in the R package “WGCNA”, PCA-PMI is available at https://github.com/Pantrick/PCA-PMI, and GRNBoost2 can be accessed at http://arboreto.readthedocs.io.

**FIGURE 4:**
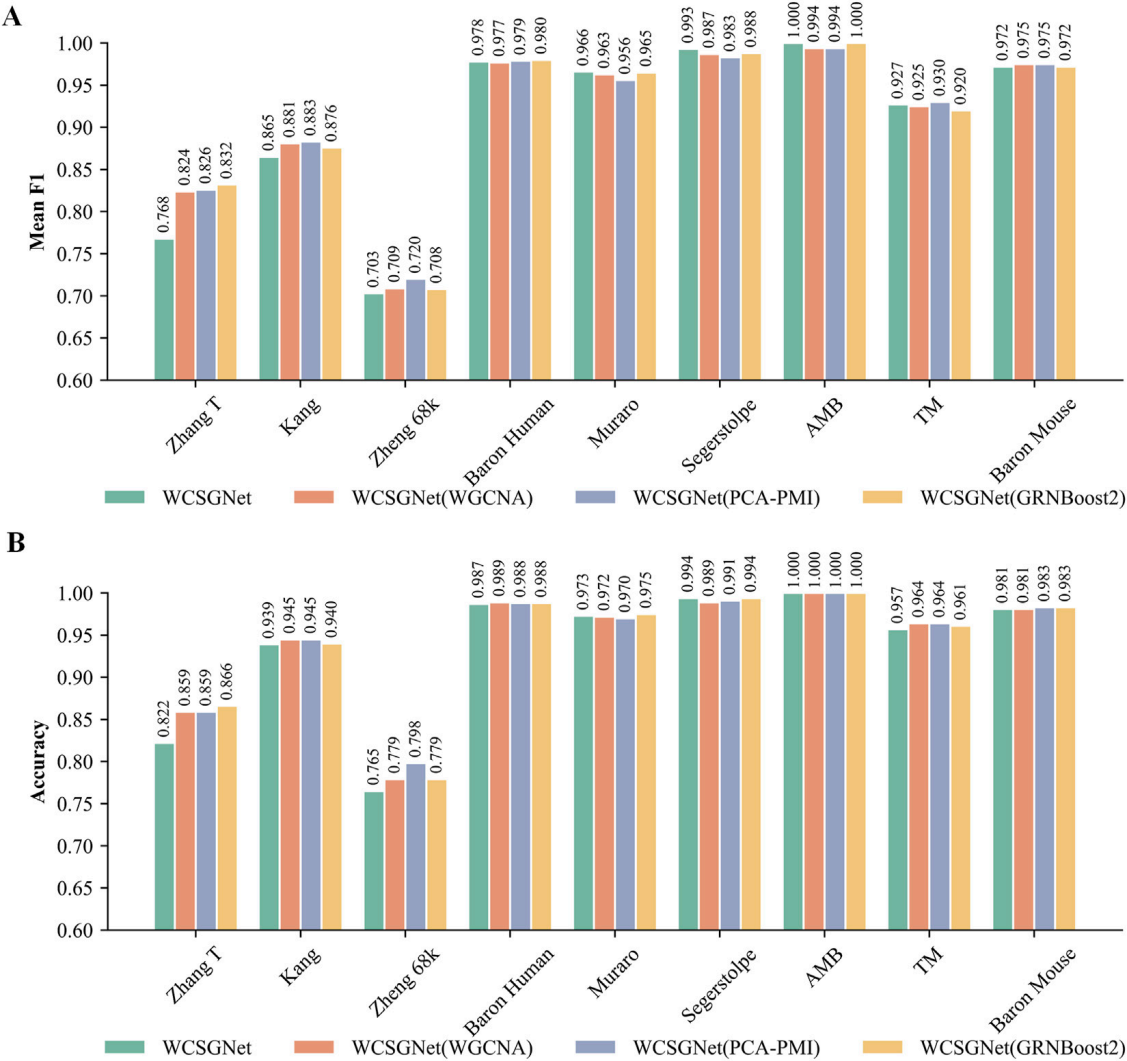
Comparison of WCSGNet performance using different gene association networks across nine scRNA-seq datasets. **(A)** Mean F1-score (bar plot) of WCSGNet using WCSN, WGCNA, PCA-PMI, and GRNBoost2 across the nine datasets. **(B)** Accuracy (bar plot) of WCSGNet using WCSN, WGCNA, PCA-PMI, and GRNBoost2 across the nine datasets.

The comparison results, presented in Figures 4A, B, show that WCSN performs similarly to other methods across the Kang, Baron Human, Muraro, Segerstolpe, AMB, TM, and Baron Mouse datasets. However, a minor performance difference is noted in the Zhang T and Zheng 68k datasets. To address this, we further analyzed and refined the weight representation method used in WCSN.

In constructing the WCSN, we used Equation 10 to assign weights for every edge in the WCSN. However, the edge weights spread to many orders of magnitude if they are assigned only in this way. Therefore, we tried to compensate this using two transformations. One is the logarithmic transformation. The other is the binary transformation. The logarithmic transformation converts each edge weight *ρ*′_*k*_ (*u*, *v*) to ln (*ρ*′_*k*_ (*u*, *v*) + 1), which is applied primarily to address the long-tailed distribution of the original edge weights. By compressing the range of these weights, the log transformation mitigates the impact of extreme values, thereby enhancing the stability and robustness of the model during both training and evaluation. Supplementary Figure S1 illustrates the distribution of edge weights before and after the logarithmic transformation for the training sets in the five-fold cross-validation across all datasets. The binary transformation assigns 1 to all edges, focusing on the network’s topological properties without considering the magnitude of the edge weights.

**FIGURE 5:**
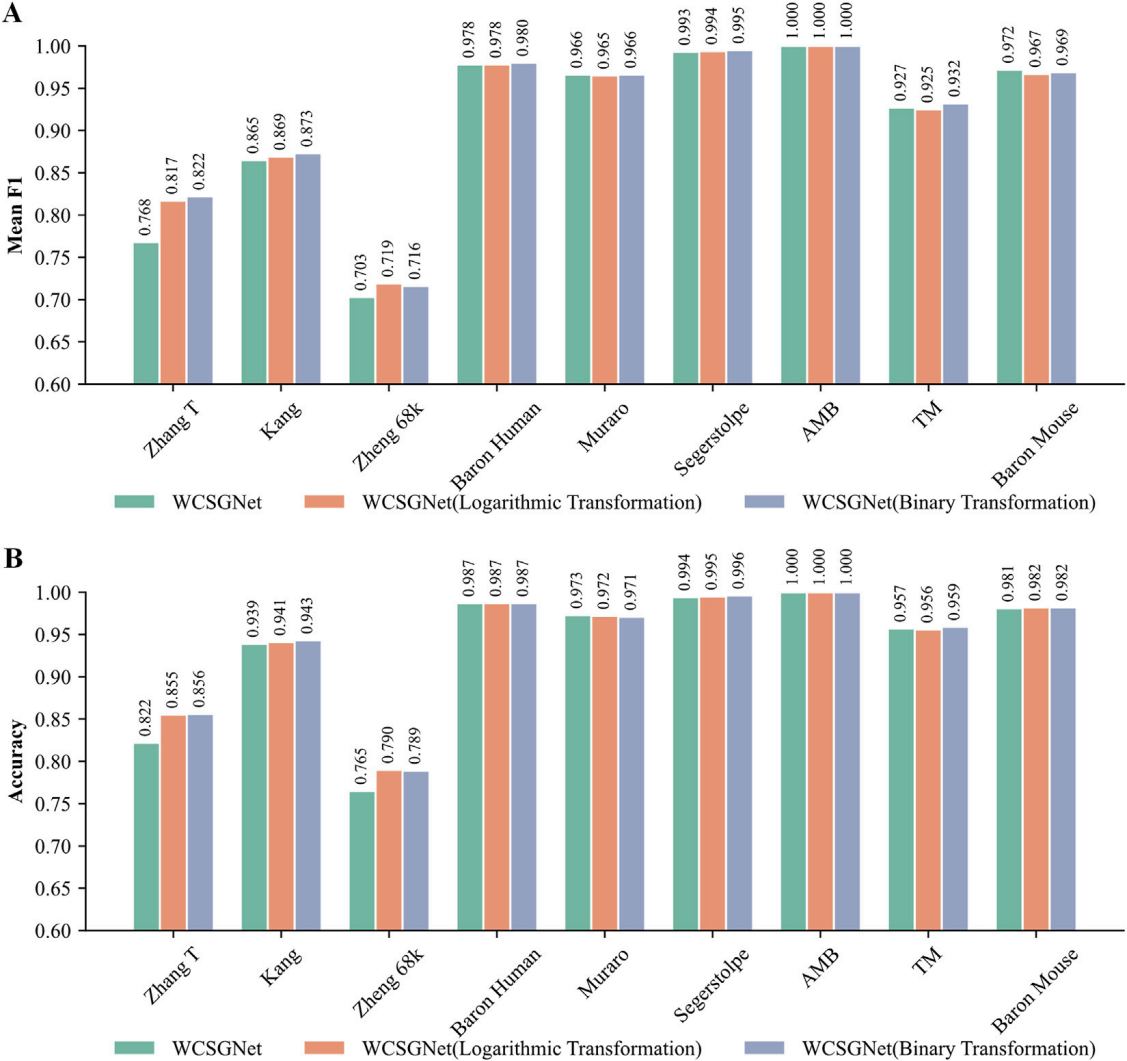
Comparison of WCSGNet performance using different edge weight representation methods on nine scRNA-seq datasets. The methods include the original method, as well as the two improved methods: logarithmic transformation and binary transformation, for WCSN construction. **(A)** Cell-type annotation performance of WCSGNet with three weight representation methods by mean F1-score (bar plot). **(B)** Cell-type annotation performance of WCSGNet with three weight representation methods by accuracy (bar plot).

We compared the performance of these three weight representations. As shown in Figures 5A, B, all three methods demonstrate consistently high mean F1-score and accuracy across all methods. However, logarithmic transformation and binary transformation achieve notable improvements over the original on the Zhang T and Zheng 68k datasets.

On the Zhang T dataset, both logarithmic transformation and binary transformation significantly outperformed the original values, with increment in mean F1-score by 6.38% and 7.03%, respectively. Similarly, accuracies improved by 4.01% and 4.14% over the original values. On the Zheng 68k dataset, logarithmic transformation and binary transformation also demonstrated superior performance, with mean F1 score improvements of 2.28% and 1.85%, respectively. Likewise, accuracies increased by 3.27% and 3.14%. Therefore, we believe these transformations improve the performance of our method.

We analyzed the sparsity of the datasets, defined as the proportion of zero elements in the count matrix (Jia et al., 2024). Among all datasets, Zheng68k exhibits the highest sparsity (Supplementary Table S4). The logarithmic transformation outperforms existing gene association network methods (WGCNA, GRNBoost2) by 1.41% and 1.55% in mean F1-score, and by 1.41% in accuracy. The binary transformation outperforms WGCNA and GRNBoost2 by 0.99% and 1.13% in mean F1-score, and by 1.28% in accuracy. These results indicate that the improved weight representation method effectively enhances performance, particularly on sparse datasets, where it demonstrates a notable advantage over other network construction methods.

### 3.3 WCSN analysis

To investigate how WCSNs contribute to the high performance of cell type classification, we focused on two key topological features: (1) hub genes, which are defined by their degree distribution, and (2) high-weight edges, which represent interaction strengths.

For each cell type, we identified top 100 hub genes and high-weight edges from the test set in each fold of the cross-validation, based on average gene degree and average edge weight. We then analyzed the consistency of WCSN structures within the same cell type across folds. Structural consistency was assessed using a coverage metric, defined as the proportion of elements (e.g., hub genes, edges) shared across all five folds, divided by the total number of elements identified in each fold. The union of the top 100 hub genes or high-weight edges from different folds was designated as the characteristic gene set or characteristic edge set, representing the key elements consistently associated with each cell type. To evaluate heterogeneity among cell types, we introduced the Uniqueness metric, which quantifies the proportion of cell type-specific elements (e.g., hub genes, edges) relative to the total number of elements in the corresponding characteristic set. This metric highlights the distinctiveness of WCSN features for each cell type.

**FIGURE 6:**
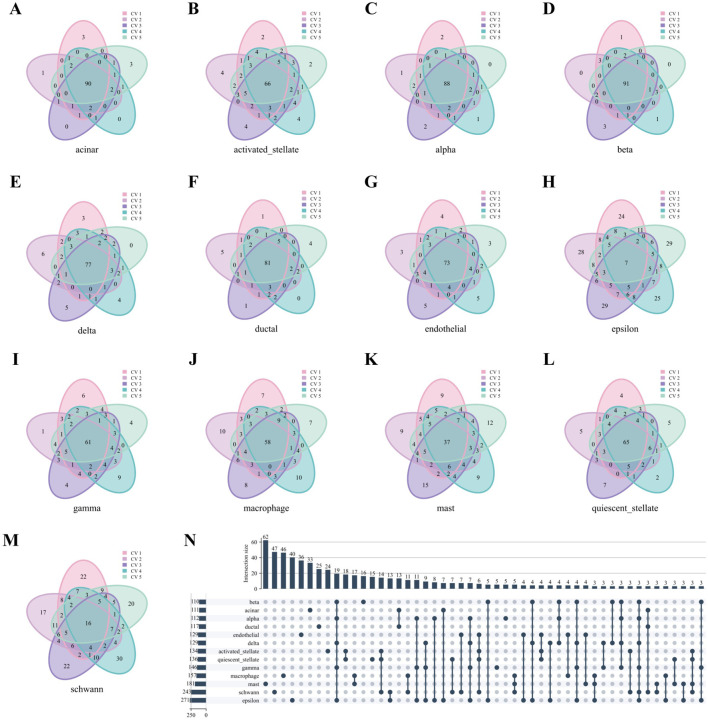
Hub genes analysis of WCSN for different cell types on the Baron Human dataset. **(A–M)** Venn diagrams showing the top 100 hub genes for 13 cell types, derived from their respective WCSNs across five folds in 5-fold cross-validation on the Baron Human dataset. Each diagram illustrates the overlap of hub genes for the corresponding cell type across the five folds. The cell type for each diagram is labeled below the plot, and the colors within the diagrams represent the individual folds of the 5-fold cross-validation. **(N)** UpSet diagram visualizing intersections of characteristic gene sets across 13 cell types, showing only intersections containing more than 2 genes. The intersections represent the overlap of gene sets across the different cell types, with only those exceeding the size threshold included. The left bar chart represents the size of each individual gene set for each cell type, while the top bar chart shows the size of each intersection, sorted by size. In the main diagram, solid dark blue-gray dots indicate the gene sets that are part of the intersection. This diagram helps identify common and unique gene sets among the cell types.

We take the Baron Human dataset as an example. As in Figures 6A–M, nearly all cell types in the Baron Human dataset demonstrated high structural consistency, as evidenced by the consistent overlap of the top 100 hub genes across all five folds. The acinar, alpha, and beta cells exhibited high coverage rates of 90%, 91%, and 88%, respectively. In contrast, the epsilon and schwann cells showed lower coverage rates of 7% and 16%, likely due to smaller sample sizes (Supplementary Table S5). However, non-unique genes from these cell types accounted for 50.18% and 54.32% of the total genes identified across all five folds, supporting the stability of their network structures despite lower coverage rates. As depicted in Figure 6N, the upset diagram demonstrated significant cell type specificity in characteristic gene sets. For instance, the acinar, beta, mast, and schwann cells displayed uniqueness values of 29.73% (33/111), 14.55% (16/110), 34.25% (62/181), and 19.34% (47/241), respectively. These findings underscore the strong cell type specificity of hub genes identified through WCSNs and highlight the importance of network topological features in distinguishing cell types.

**FIGURE 7:**
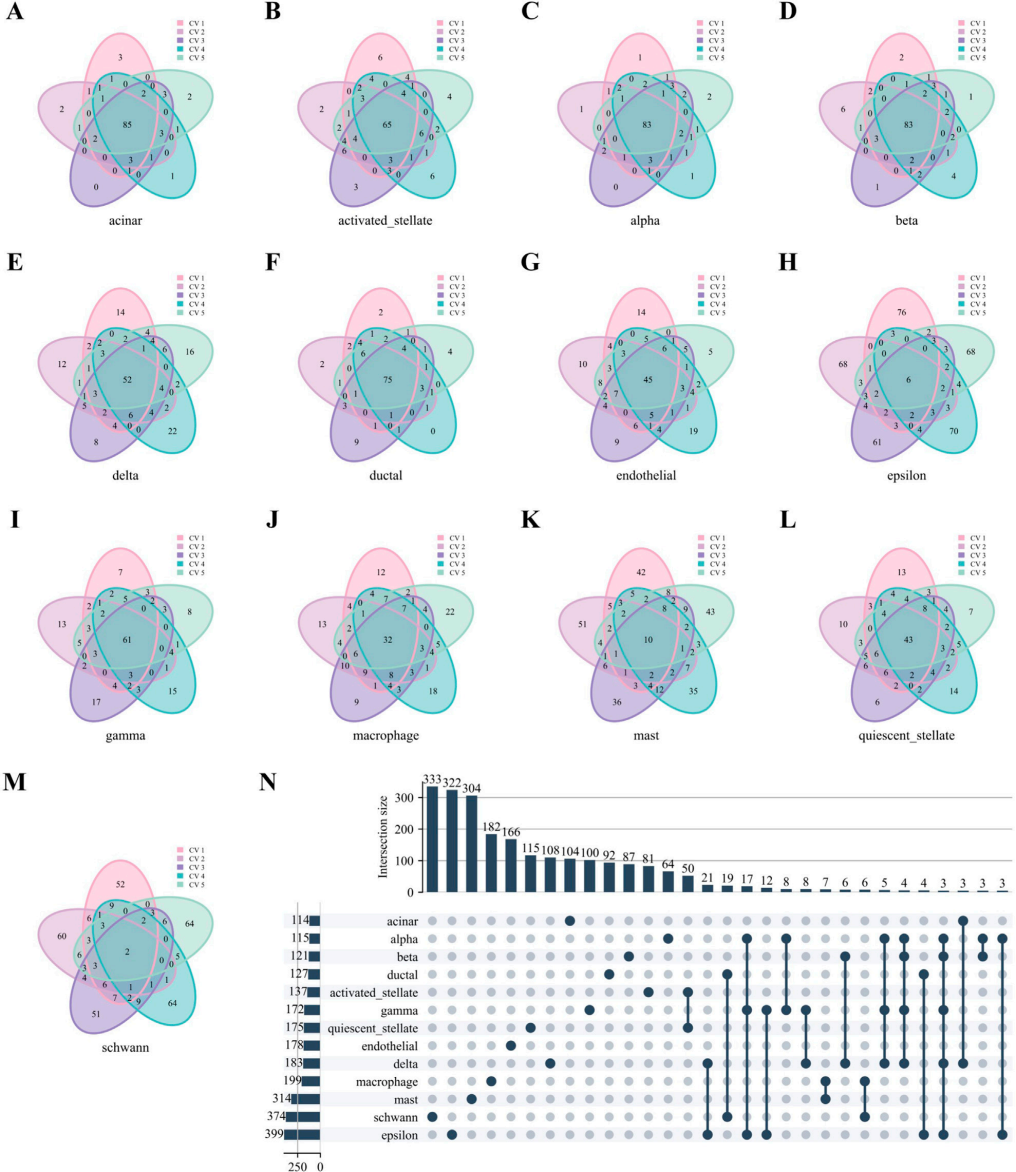
High-weight edges analysis of WCSNs for different cell types in the Baron Human dataset. **(A–M)** Venn diagrams display the top 100 high-weight edges for 13 cell types, derived from their respective WCSNs across five folds in 5-fold cross-validation on the Baron Human dataset. Each diagram illustrates the overlap of high-weight edges for the corresponding cell type across the five folds. The cell type for each diagram is labeled below the plot, and different colors within the diagrams represent the individual folds of the 5-fold cross-validation. **(N)** The UpSet diagram presents the intersections of characteristic edge sets across cell types, with intersection size threshold >2. The intersections represent the overlap of edges sets across the different cell types, with only those exceeding the size threshold included. The left bar chart represents the size of each individual edge set for each cell type, while the top bar chart shows the size of each intersection, sorted by size. In the main diagram, solid dark blue-gray dots indicate the edge sets that are part of the intersection. This diagram helps identify common and unique edge sets among the cell types.

Similarly, Figures 7A–M reveals robust stability in high-weight edges across most cell types in the Baron Human dataset, paralleling the previously observed stability of hub genes. Notably, high-weight edges showed distinct, mutually exclusive distributions among different cell types (Figure 7N).

**FIGURE 8:**
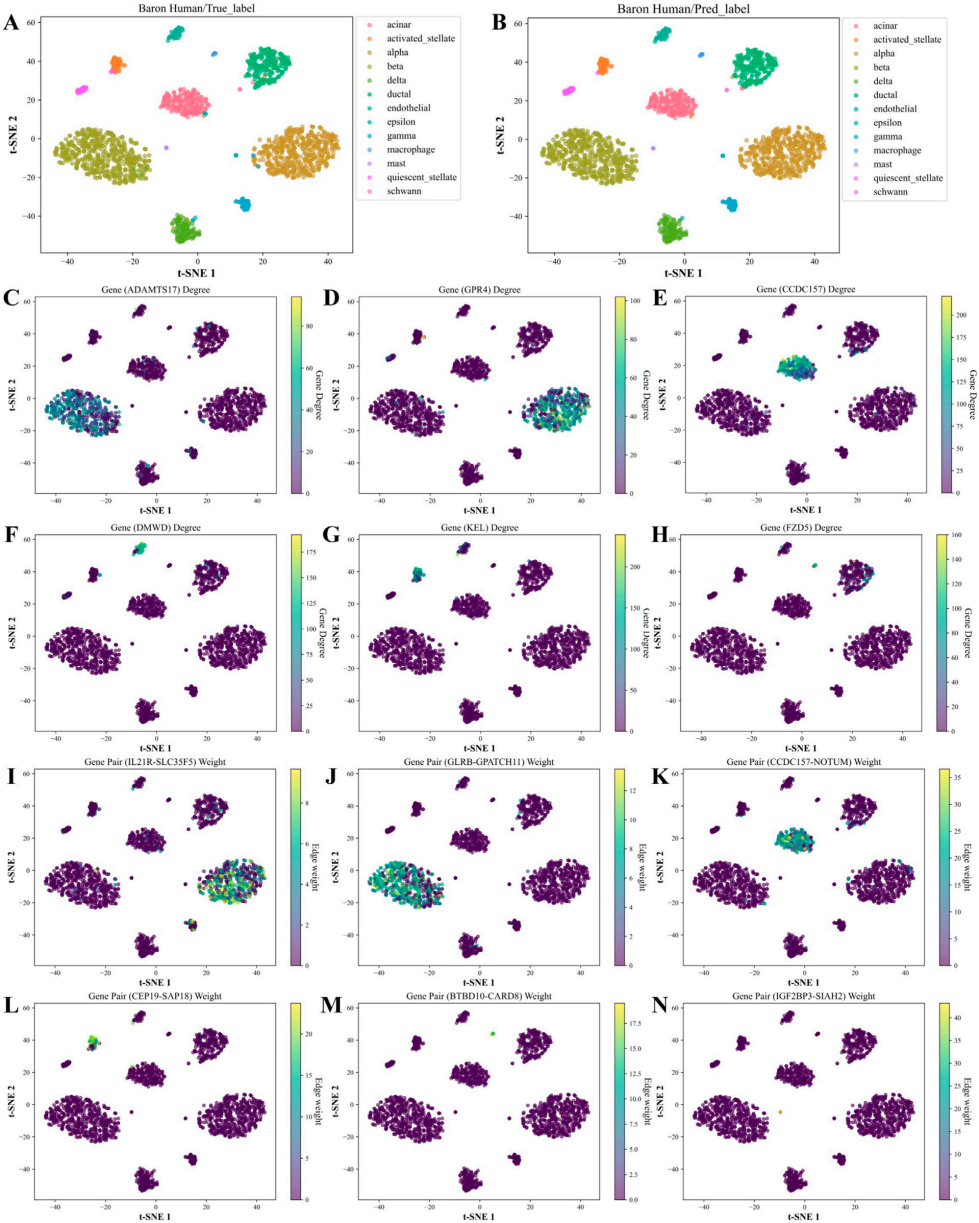
*t*-SNE visualization and feature analysis of the Baron Human dataset using WCSGNet. **(A, B)** *t*-SNE visualization of high-level features extracted by WCSGNet, colored by **(A)** true cell types and **(B)** predicted cell types. **(C)** Visualization of ADAMTS17, a top hub gene uniquely identified in the characteristic gene set of beta cells, with *t*-SNE coloring representing its degree in WCSNs. **(D–H)** Visualizations of five additional hub genes, showcasing cell type-specific connectivity patterns. The top titles of each plot include the gene names, with each plot representing specific gene connectivity patterns in (alpha, acinar, endothelial, activated stellate, macrophage) cell types. **(I)** Visualization of IL21R-SLC35F5, a top high-weight edge uniquely identified in the characteristic edge set of alpha cells, with *t*-SNE coloring indicating its interaction strength in WCSNs. **(J–N)** Visualizations of five additional high-weight edges, highlighting cell type-specific interaction patterns across different cell types. The top titles of each plot include the gene pairs, with each plot depicting interaction patterns specific to (beta, acinar, activated stellate, macrophage, mast) cell types.

Based on the above analysis, we generated the hub genes and high-weight edges for each cell type (Supplementary Tables S6, S7). To illustrate the role of cell type-specific hub genes and high-weight edges in cell type annotation, we applied t-SNE on the Baron Human dataset for visualization (Figure 8). As in Figures 8C–H, unique hub genes for each cell type clearly distinguish the corresponding cell types. Likewise, unique high-weight edges significantly contribute to cell type classification (Figures 8I–N).

**FIGURE 9:**
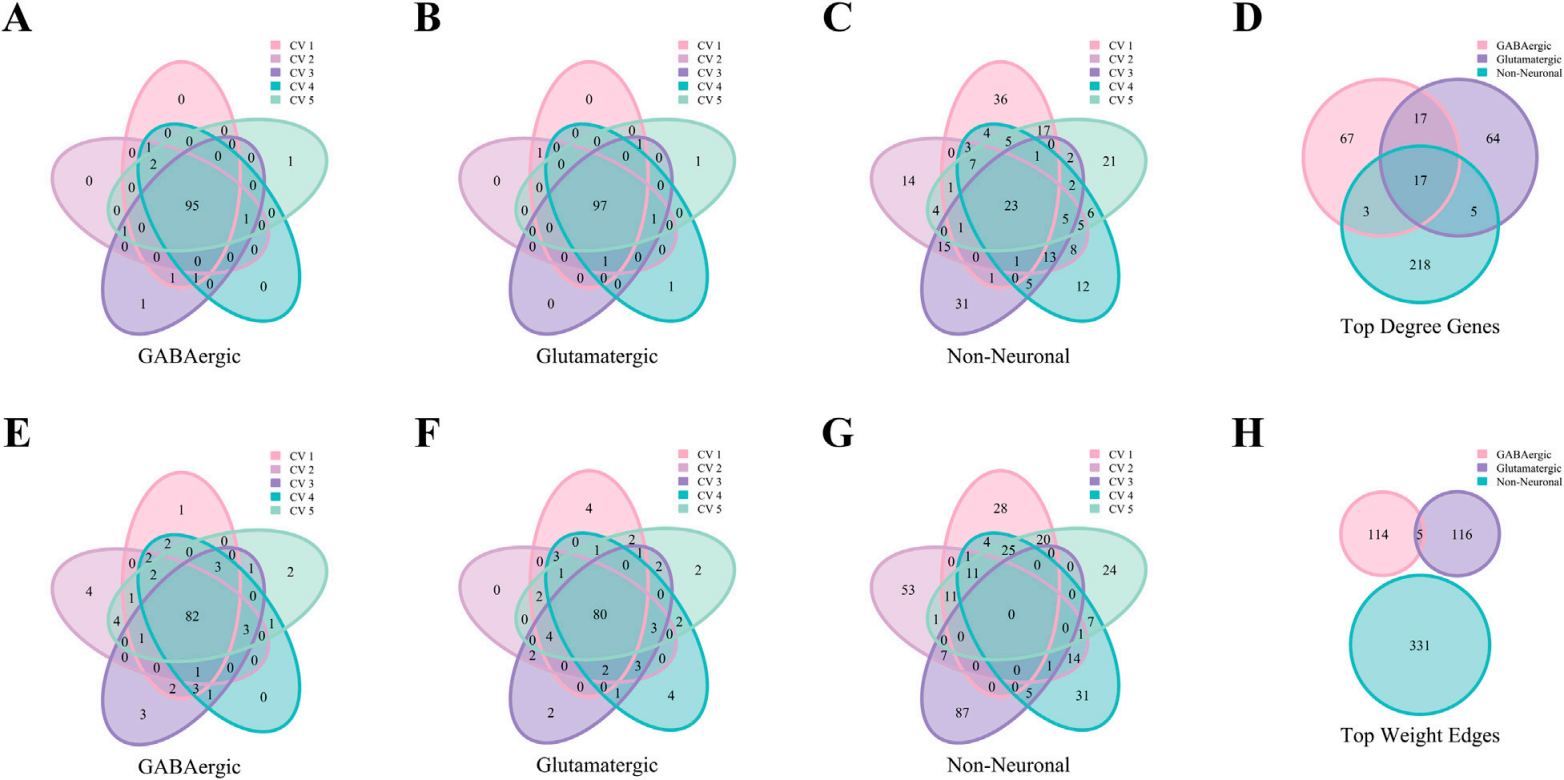
Analysis of hub genes and high-weight edges in WCSN for different cell types on the AMB Dataset. **(A–C)** Venn diagrams showing the top 100 hub genes for GABAergic, Glutamatergic, and Non-Neuronal cell types across five folds in 5-fold cross-validation. Each diagram highlights the overlap of top 100 hub genes within the respective cell type, with colors representing the individual folds of the 5-fold cross-validation. **(D)** Venn diagram showing the characteristic gene sets for the 3 cell types, emphasizing shared and unique hub genes. Colors represent the respective cell types. **(E–G)** Venn diagrams illustrating the top 100 high-weight edges for GABAergic, Glutamatergic, and Non-Neuronal cell types across five folds in 5-fold cross-validation. Each diagram represents the overlap of high-weight edges for the corresponding cell type across the five folds, with colors indicating the individual folds. **(H)** Venn diagram presenting the characteristic edge set across five folds for the 3 cell types, highlighting the unique gene pairs with strong interactions specific to each cell type. Colors represent the cell types.

Figure 9 demonstrates that the AMB dataset exhibits patterns that are in consistent with the Baron Human dataset. This figure highlights the consistency of top hub genes and high-weight edges across folds, as well as the specificity of characteristic gene and edge sets for various cell types. These results confirm that key patterns identified in the human dataset are conserved in the mouse dataset, further validating the robustness and generalizability of the analytical approach.

Our analysis demonstrates that both hub gene degree and interaction strength in WCSNs are biologically meaningful and critical for distinguishing cell types. The stability of these features across folds within the same cell type, as well as their specificity across different cell types, highlights their robustness in capturing cell-specific regulatory patterns. These findings provide strong evidence that WCSGNet leveraging WCSNs, is an effective tool for cell type classification and offers novel insights into the molecular mechanisms underlying cellular heterogeneity.

## 4 Discussions

In construction of the WCSN in this study, we primarily followed the methodology outlined in the CSN paper. In this approach, the settings for R_*u*_ and R_*v*_ are based on a fixed ratio of the total number of cells, simplifying the network construction. An alternative approach is to set R_*u*_ and R_*v*_ as a fixed ratio of the overall expression range of the *u*-th gene and *v*-th gene, which could offer more biological relevance. Future studies could explore and potentially improve upon this approach.

To address class imbalance among cell types, we implemented up-sampling in the training dataset. However, this method carries some risks, such as overfitting, since the random duplication of samples may lead the model to rely too heavily on repeated instances, particularly for rare cell types. Furthermore, up-sampling does not introduce novel information, which limiting the diversity and variability of the rare cell types. To overcome these limitations, future research could investigate advanced techniques such as the Synthetic Minority Over-sampling Technique (SMOTE) (Chawla et al., 2002), which generates synthetic samples to increase cell type diversity while mitigating overfitting risks. Additionally, we employed a weighted cross-entropy loss function to address class imbalance by assigning higher weights to rare cell types. Although effective, this method may inadvertently overemphasize the rare cell types, increasing the risk of overfitting. Future work should refine these strategies to better balance class representation and generalizability.

Furthermore, the network construction method used in WCSGNet still has potential for performance improvement. Future research will focus on further enhancing both the network construction and weight representation methods to improve the network’s stability and biological relevance, allowing for more effective handling of sparse datasets.

## 5 Conclusion

We developed WCSGNet, an innovative approach for cell type annotation using scRNA-seq data. WCSGNet generates weighted, cell-specific gene association networks for individual cells and employs graph neural networks to extract informative features. Comparative analyses demonstrate that WCSGNet achieves comparable or better performances than state-of-the-art methods. WCSGNet integrates gene expression features with cell-specific network features for cell representations. This opens a new way for cell representations based on scRNA-seq data. We anticipate that WCSGNet will serve as a valuable tool for automated cell type annotation.

## Data Availability

Publicly available datasets were analyzed in this study. This data can be found here: https://github.com/Yi-ellen/WCSGNet.
