# Supplementary figures and images for: WCSGNet: a graph neural network approach using weighted cell-specific networks for cell-type annotation in scRNA-seq
Source: Front Genet. 2025 Feb 17;16:1553352. doi: 10.3389/fgene.2025.1553352 (PMC11872911; doi:10.3389/fgene.2025.1553352)

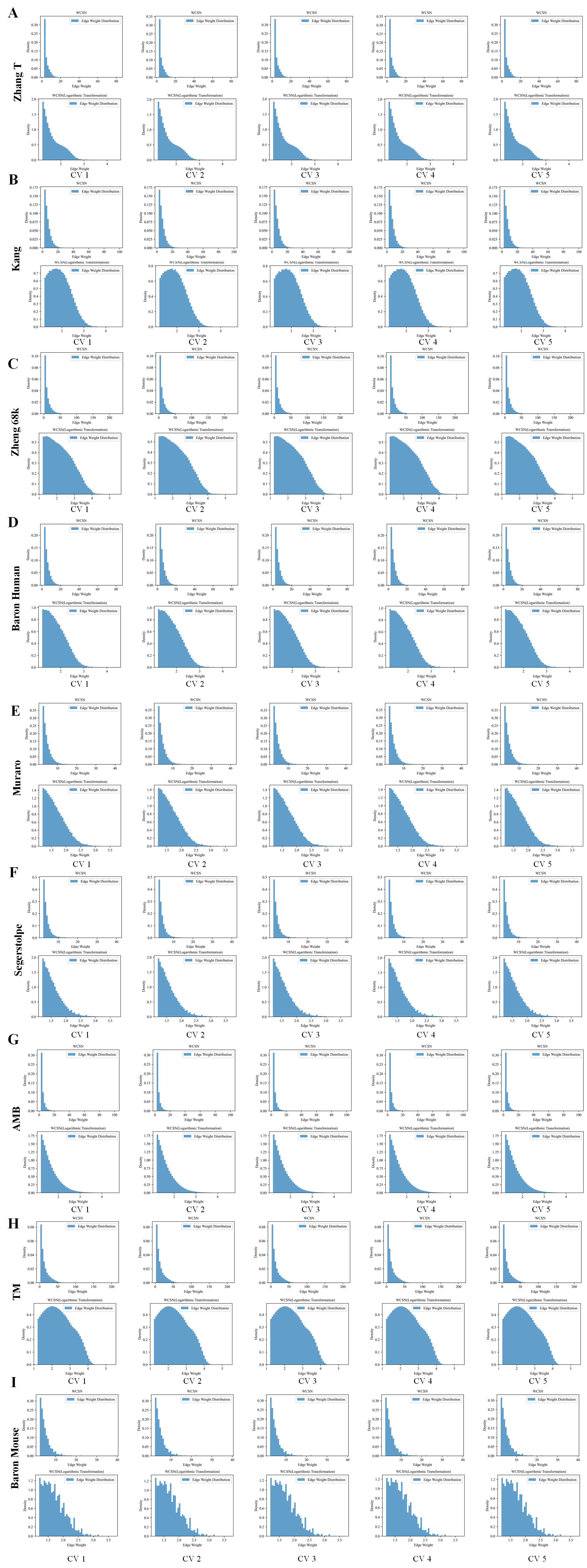

Supplement: Supplementary file 1 [file Image1.tif]
