# Supplementary material for: WCSGNet: a graph neural network approach using weighted cell-specific networks for cell-type annotation in scRNA-seq
Source: Front Genet. 2025 Feb 17;16:1553352. doi: 10.3389/fgene.2025.1553352 (PMC11872911; doi:10.3389/fgene.2025.1553352)
Supplement: Supplementary file 2 [file Table2.docx]

Supplementary Material

# Supplementary Figures

**Figure S1.** Distribution of edge weights before and after logarithmic transformation for the training sets in five-fold cross-validation across all datasets including Zhang T, Kang, Zheng 68k, Baron Human, Muraro, Segerstolpe, AMB, TM and Baron Mouse.
